# Adenocarcinoma Arising From a Gastric Duplication Cyst With Lymph Node Metastasis

**DOI:** 10.7759/cureus.12320

**Published:** 2020-12-27

**Authors:** Shoichi Kinugasa, Hiroyuki Monma, Yoshio Sakamoto, Takafumi Watanabe, Masayo Fujimoto

**Affiliations:** 1 Surgery, Hyogo Prefectural Kakogawa Medical Center, Kakogawa, JPN; 2 Gastroenterology, Hyogo Prefectural Kakogawa Medical Center, Kakogawa, JPN; 3 Pathology, Hyogo Prefectural Kakogawa Medical Center, Kakogawa, JPN

**Keywords:** gastric duplication cyst, adenocarcinoma, malignancy, gastrectomy, surgery

## Abstract

Gastric duplication cysts (GDCs) are a relatively rare congenital anomalies and are mostly diagnosed in the early years of life. Herein, we report a very rare surgical case of adenocarcinoma arising from a GDC with lymph node metastasis. A 78-year-old woman was referred to our hospital because of elevated serum levels of cancer antigen (CA) 19-9. Endoscopic ultrasound, contrast fistulography, and computed tomography showed a cystic lesion communicating with the lesser curvature of the stomach. The serum levels of CA 19-9 were high, and fluorine-18 fluorodeoxyglucose positron emission tomography/computed tomography (18F-FDG PET/CT) imaging demonstrated a slightly enlarged lymph node with high FDG uptake after four months. The size of the cyst was unchanged. It was diagnosed as a GDC. The enlarged lymph node was highly likely to be malignant. Hence, we performed a distal gastrectomy involving the origin of entry and whole body of the GDC with en bloc regional lymphadenectomy. The postoperative pathology was consistent with GDC with moderately differentiated adenocarcinoma and lymph node metastasis. Adjuvant chemotherapy with tegafur-gimeracil-oteracil potassium (S-1) was administered for 12 months. At present, the patient is alive, with no recurrence of the lesion even four years after the operation. GDCs in adults are rare and may predispose to malignancy. Early diagnosis and prompt surgical intervention are important for favorable outcomes.

## Introduction

Gastric duplication cysts (GDCs) are relatively rare congenital anomalies that are mostly diagnosed in the early years of life. It is usually diagnosed before age 12. GDCs in adults are generally detected incidentally. This incidental detection is also because most cases are asymptomatic though in some cases, a symptomatic obstruction may occur. One-third of GDCs are associated with other developmental anomalies such as GDCs at other locations and duodenal diverticula [1]. Adenocarcinoma rarely arises from a GDC, and only 16 cases of malignancy have been reported to date [2-16]. An established resection procedure for and the range of this malignancy have not been discovered thus far. We present a case of a patient showing long-term survival after gastrectomy and lymph node dissection for adenocarcinoma arising from a GDC with a metastatic lymph node at the lesser curvature of the stomach.

## Case presentation

**Figure 1:**
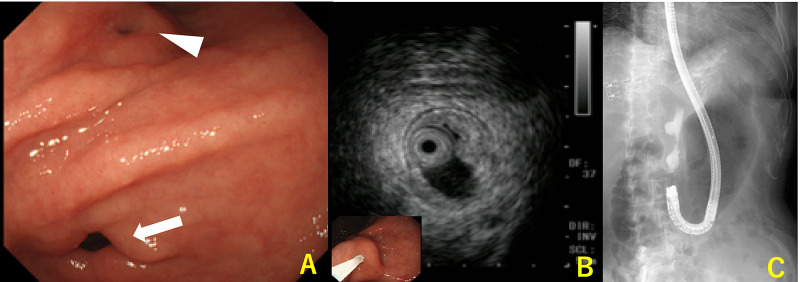
Pre-operative images Upper gastrointestinal endoscopy shows a small orifice along the lesser curvature of the lower body of the stomach; the pyloric ring (arrow) and orifice of the duplication cyst (arrowhead) are seen (A). Endoscopic ultrasound shows a tubular lesion lined by a mucosal layer with the surrounding muscularis propria visible in the root of the gastric cyst, suggestive of a duplication cyst (B). Contrast fistulography shows a tubular lesion, 7 cm in length, with a stricture in the middle portion of the lesion (C).

**Figure 2:**
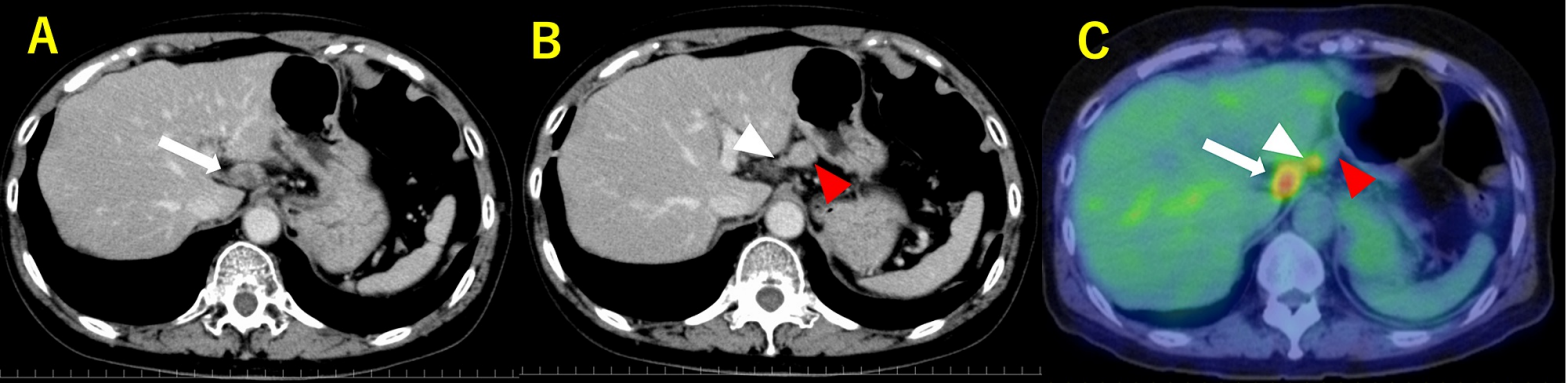
CT and PET/CT Pre-operative enhanced computed tomography (A,B) and Fluorine-18 fluorodeoxyglucose (FDG) positron emission tomography/computed tomography imaging (C) demonstrates a slightly enlarged lymph node with high FDG uptake (SUVmax =5.6, white arrow). The size of the cyst (red arrowhead) was unchanged. FDG uptake (SUVmax =3.9, white arrowhead) showed in stricture region.

**Figure 3:**
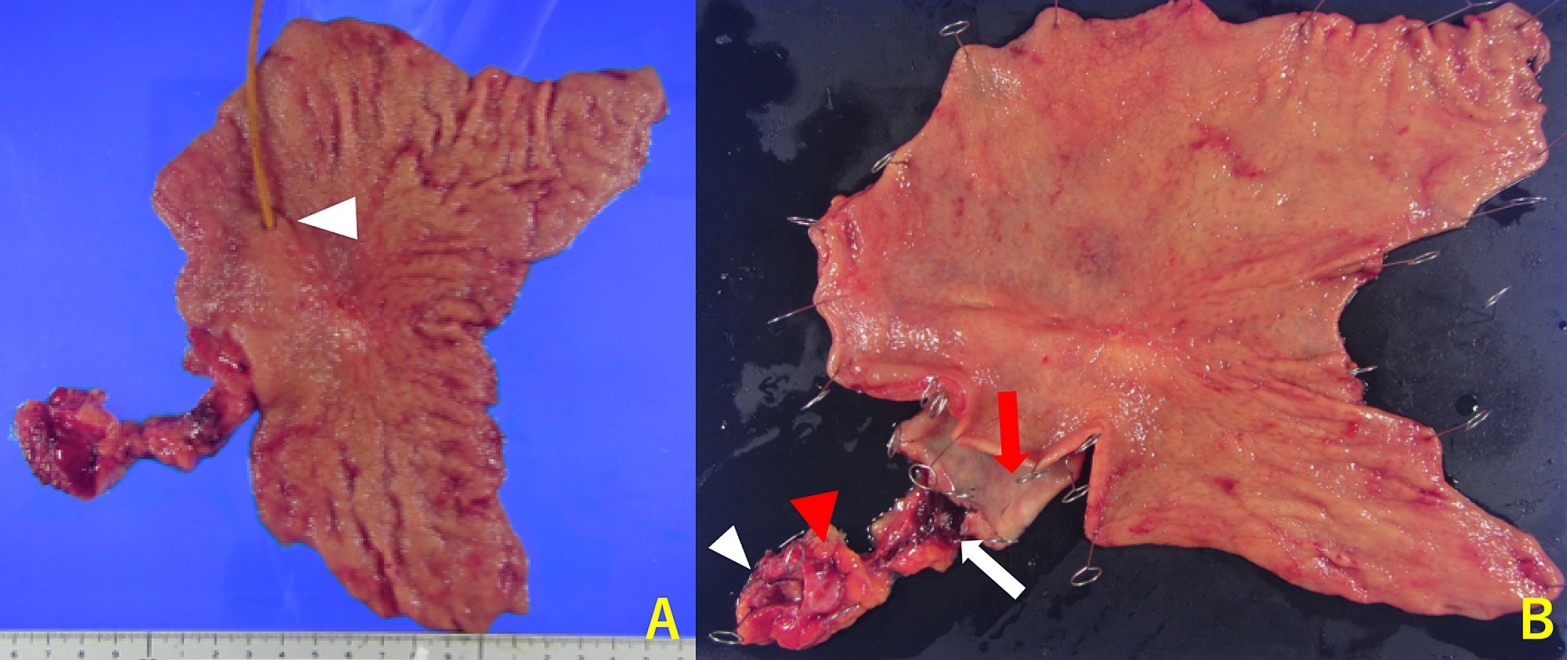
Resected specimen Resected specimen: the orifice of the duplication cyst (white arrowhead) (A); macroscopic view: the mucosal surface of the duplication cyst (red arrow), stricture revealing the cancer cells (white arrow), blind end of the duplication cyst (red arrowhead), metastatic lymph node (white arrowhead) (B).

**Figure 4:**
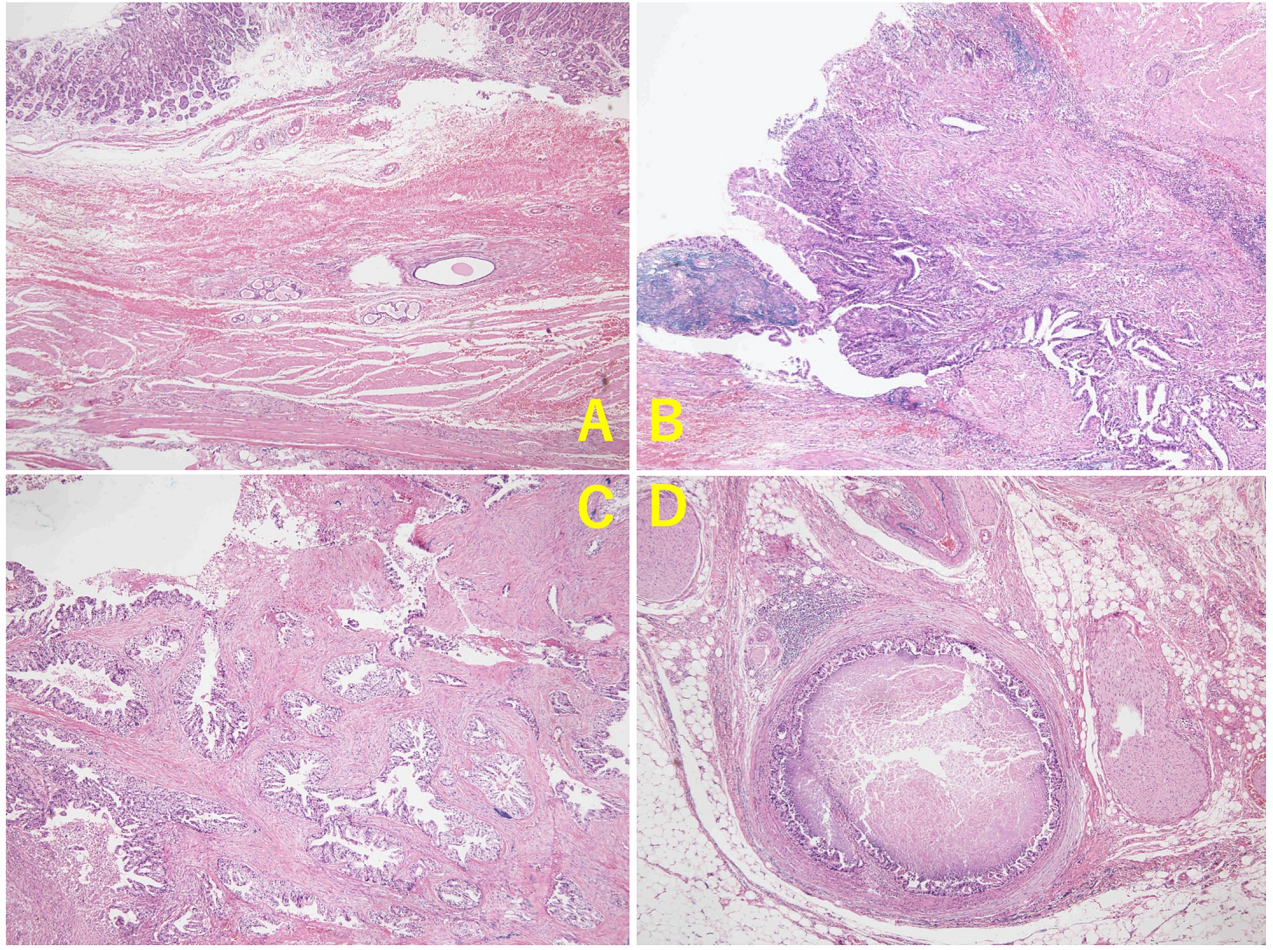
Microscopic findings of a gastric duplication cyst The gastric side of the cystic wall has an inner mucosal lining, the submucosa, and muscularis propria contiguous with that of the gastric wall (A). Moderately differentiated adenocarcinoma confined to the mucosal layer is mainly observed in the proximal part of the duplication cyst (B); deep infiltration by the cancer cells; the layered structure of the cystic wall disappears in the distal part of the duplication cyst (C); vascular invasion (D).

A 78-year-old woman was referred to our hospital because of incidentally elevated serum levels of cancer antigen (CA) 19-9 by medical check including cancer screening with no significant clinical symptoms. She had a history of hypertension, but she had no other significant past history nor family history of malignancy. She was diagnosed with a *Helicobacter pylori* infection by a stool antigen test and subsequently underwent eradication therapy just before her visit to our hospital and it was successful. All laboratory test results were normal except the CA 19-9 concentration, which was 186.9 U/mL (normal, <37 U/mL). The patient underwent serial diagnostic imaging examinations of the alimentary system. Upper gastrointestinal endoscopy revealed a small orifice in the lesser curvature of the lower body of the stomach (Figure 1A). Suction cytology from the hole did not detect any malignant cells. Endoscopic ultrasound (EUS) and contrast fistulography through the hole showed a tubular lesion lined by a mucosal layer contiguous with that of the stomach, and the surrounding muscularis propria was visible in the root of the GDC (Figure 1B, 1C). In the distal portion of the cyst, the wall of the GDC lacked the mucosal layer and muscularis propria. Abdominal computed tomography (CT) showed a cystic lesion along the lesser curvature of the stomach and lymph node swelling. We followed up these lesions using CT because the patient did not report any discomfort. The serum levels of CA 19-9 increased to 512.4 U/mL four months later. Fluorine-18 fluorodeoxyglucose positron emission tomography/CT (18F-FDG PET/CT) imaging demonstrated a slightly enlarged lymph node with high FDG uptake (maximum standardized uptake value [SUVmax] = 5.6). The size of the cyst was unchanged (SUVmax = 3.9) (Figure 2). It was determined that the GDC and enlarged lymph node were highly likely to be malignant. Resection of the primary lesion with negative surgical margins and lymphadenectomy were considered necessary. Laparotomy revealed a cystic mass that originated from the angle of the lesser curvature of the stomach and was growing toward the liver. A swollen hard lymph node, regional right cardiac node, was located at the tip of the cystic lesion adjacent to the liver. The peritoneal lavage fluid that was collected and examined immediately after laparotomy was found to be cytology negative. We performed a distal gastrectomy involving the orifice and entire body of the GDC (11 cm in length), with en bloc D2 regional lymphadenectomy and Billroth 1 reconstruction, and the patient was discharged two weeks after the surgery (Figure 3). The postoperative pathology was consistent with GDC with moderately differentiated adenocarcinoma and a lymph node metastasis out of 34 dissected nodes with lymphatic and blood vessel invasion. The cancer cells were confined to the mucosa of the root of the GDC. In the distal part of the GDC, the cancer cells were present in the damaged layered structure of the GDC, and the depth of the tumor was determined as T3 (Figure 4). The final tumor-node-metastasis stage was pT3N1M0, stage IIB. Adjuvant chemotherapy with tegafur-gimeracil-oteracil potassium (S-1) was administered for 12 months. The patient has been followed up at our hospital every six months with CT in combination with measurement of CA-19 and annual endoscopy. At present, the patient is alive without any recurrence of the lesion even four years after the operation.

## Discussion

This report reveals that GDCs in adults are rare and may predispose to malignancy. Early diagnosis and prompt surgical intervention are important for favorable outcomes. The term ‘alimentary tract duplication’ was used by Ladd to describe congenital malformations that involve the mesenteric side of the associated alimentary tract and share a common blood supply with the native bowel [17]. Alimentary tract duplications are rare and occur anywhere along the digestive tract in one in 4500 births [18]. The most common locations of duplication are the small intestine, esophagus, and colon. GDCs represent 4% of all duplication cysts [15]. The typical features of GDCs are as follows: a) the cyst wall is contiguous with the stomach wall; (b) the cyst is surrounded by smooth muscle, which is contiguous with the muscle of the stomach; and (c) the cyst wall is lined by the epithelium of the alimentary tract. The lesions found in our case fulfilled these criteria for GDCs. Most cases of GDC are diagnosed in the first year of life and with vomiting and a palpable abdominal mass. It is unusual for GDCs to remain asymptomatic until adulthood, and the patient in our case is one of the oldest GDC patients to be diagnosed in adulthood to date.

GDCs are classified as either cystic or tubular depending on their communication and contiguity with the stomach. More than 80% of gastric duplications are cystic and do not communicate with the gastric lumen. The remaining 20% comprise tubular GDCs, which are contiguous with the stomach and communicate to a certain extent with its lumen [13]. Duplication cysts of the ileum are usually located on the mesenteric border, whereas the usual location for GDCs is along the greater curvature, followed by the posterior wall of the stomach. This is because these duplications are usually distributed dorsal to the primitive gut during development. GDCs are located along the lesser curvature only in 5.5% of reported cases, and the mechanism by which they develop is not well understood [19]. In our case, the tubular-shaped GDC was located along the lesser curvature of the stomach and communicated with the gastric lumen. Within the root of the GDC, the lumen was surrounded by the gastric mucosa and smooth muscle. 

Adenocarcinoma arising from a GDC is extremely rare with only 16 cases reported in the literature (Table 1) [2-16]. As shown in Table 1, carcinomas arising from GDCs generally occur in middle-aged adults and are diagnosed as large-sized masses. The probability of malignant transformation of GDCs increases with age. Persistent exposure to carcinogenic substances such as salty pickles and *Helicobacter pylori* infection is considered to be the cause of malignant transformation. However, almost all adenocarcinomas arising from GDCs are cystic, without communication with the gastric lumen. This case is the first report of adenocarcinoma originating from a GDC and communicating with the lesser curvature of the stomach. The patient was diagnosed with a *Helicobacter pylori* infection by a stool antigen test and subsequently underwent eradication therapy. *Helicobacter pylori*-induced gastritis may have led to gastric cancer in this case. Up to 10% of GDCs may contain ectopic pancreatic tissue, which may lead to pancreatitis that mimics a pancreatic pseudocyst. However, malignant transformation of heterotopic pancreatic tissue is extremely rare. Persistent irritation together with an increase in the intracystic pressure and oxygen deficiency may cause chronic inflammation, repeated apoptosis, and regeneration of the epithelium, which may ultimately lead to malignant transformation within the GDC [11]. In this situation, complications such as obstruction, necrosis, and infection were possible, but the patient did not have these complications until adulthood.

**Table 1:** Clinicopathological data of patients diagnosed with carcinoma arising from a gastric duplication cyst. NA: not available; GC: greater curvature; LC: lesser curvature; WNL: within normal limits; tub1: well-differentiated adenocarcinoma; tub2: moderately differentiated adenocarcinoma; muc: mucinous carcinoma; pap: papillary adenocarcinoma; ADC: adenocarcinoma; NEC: neuroendocrine carcinoma; SCC: squamous cell carcinoma; DPS: distal pancreatosplenectomy; H: hepatectomy; PD: pancreaticoduodenectomy; DP: distal pancreatectomy; mo: month; y: year

Reference	Age	Sex	Symptoms	Location	Communication	CA19-9(U/ml)	Histology	Size	Surgery	Outcome
Mayo et al. [2]	64	F	Weakness, anorexia	GC/Antrum	No	NA	tub1	6cm	Distal gastrectomy	12m/Alive
Coit et al. [3]	72	F	Pain ,weight loss	GC	No	NA	muc/pap	3.2cm	Distal gastrectomy	72m/Alive
Kuraoka et al. [4]	40	M	Fever, backpain	GC	No	NA	ADC	7cm	Proximal gastrectomy	7m/Alive with recurrence
Horne et al. [5]	40	M	Pain	GC	No	NA	NEC	12cm	Total gastrectomy+DPS+H	14m/Alive with recurrence
Barussaud et al. [6]	67	F	Mass, weight loss	Antrum	NA	280	ADC/SCC	18cm	Distal gastrectomy	6m/Dead
Fukumoto et al. [7]	50	M	Vomiting	Duodenum	No	297	tub1/por	3cm	Local Excision, PD	Dead
Zheng et al. [8]	25	M	Asymptomatic	GC	No	NA	ADC	8cm	Total gastrectomy	13m/Alive
Kang et al. [9]	56	M	Asymptomatic	GC/Corpus	No	NA	ADC	5.5cm	Local Excision	NA
Liu et al. [10]	28	M	Asymptomatic	GC/Corpus	No	2145	ADC	13cm	Local Excision	7m/Alive with metastasis
Zhu et al. [11]	62	M	Pain	GC	No	NA	NEC	4cm	Total gastrectomy	NA
	72	M	Regurgitation	Posterior	No	NA	NEC	2cm	Distal gastrectomy	NA
Yamasaki et al. [12]	47	F	Asymptomatic	GC	No	167.1	tub2	10cm	Local Excision	9m/Dead
Abdulla et al. [13]	51	M	Melena	GC	Yes	NA	tub2	5cm	Total gastrectomy	NA
Han et al. [14]	41	F	NA	Posterior	No	NA	tub2/SCC	6cm	Total gastrectomy+DP	NA
Sethi et al. [15]	63	M	Vomiting	Antrum	No	WNL	pap	10cm	Distal gastrectomy+H	NA
Chang et al. [16]	57	F	Abdominal discomfort	Posterior	No	WNL	tub2	8.2	Total gastrectomy	6m/Alive
Present case	72	F	Asymptomatic	LC	Yes	512.38	tub2	11cm	Distal gastrectomy	4y/Alive

Gastritis cystica profunda (GCP) is one of the most important differential diagnoses. GCP usually develops in the stomach after gastrectomy and is considered to be a precancerous lesion associated with Epstein-Barr virus infection. Adenocarcinoma rarely arises from GCP in the unoperated stomach. The difference between adenocarcinoma arising from a GDC and that arising from GCP is the presence of a smooth muscle coat surrounding the lesion in case of the former [9]. A gastrointestinal stromal tumor (GIST) is also an important differential diagnosis because the stomach is a common location for GISTs. GISTs usually present as solid tumors; however, sometimes large areas of internal hemorrhage and cystic degeneration may occur [14]. CT is useful in detecting the lesion and determining its relationship with other organs. EUS and EUS-guided fine-needle aspiration can provide a more definitive diagnosis. PET/CT was also useful in distinguishing between benign and malignant lesions in this case. Estimating serum CA 19-9 levels may be useful in predicting malignancy in some cystic lesions such as ovarian cysts or intraductal papillary mucinous neoplasms of the pancreas. In gastric cancer, elevated serum CA 19-9 levels are predictive of lymph node metastasis [20]. Among the reviewed cases, including the present case, the serum CA 19-9 levels were elevated in five cases. Barring our case, the four remaining cases showed early recurrence and a poor prognosis. In gastric cancer originating from GDCs, the depth of invasion is not always associated with the size of the GDCs. In almost all reported cases, stomach resection surgery was performed. Moreover, the long-term prognosis after local excision of the GDC has not been confirmed. GDCs in adults, especially lesions with elevated serum CA 19-9 levels, should be managed surgically via gastric resection and regional lymphadenectomy [7].

This is the only case in which the long-term prognosis over a four-year-period has been reported after surgery for gastric cancer with lymph node metastasis arising from a GDC. In most cases, tumors without communication with the gastric lumen grow asymptomatically. By the time symptoms appear, such tumors have already invaded the surrounding organs or metastasized. Although the diagnosis of malignancy originating from a GDC is difficult, once a GDC is detected, even if it is asymptomatic, it should be removed, considering its potential for malignancy. We should be careful not to rupture the cyst wall as this will result in peritoneal carcinomatosis.

## Conclusions

In conclusion, GDCs are rare and usually present as benign lesions but may predispose to malignancy and other complications in adults. GDCs should be considered in the differentiation of cystic masses of the gastrointestinal tract. Once detected, prompt surgical intervention is important for optimal outcomes.
